# Cytokine expression profiles in children and adolescents with tic disorders

**DOI:** 10.1038/s41598-024-62121-z

**Published:** 2024-07-02

**Authors:** Meryem Ozlem Kutuk, Ali Evren Tufan, Fethiye Kilicaslan, Cem Gokcen, Gulen Guler Aksu, Cigdem Yektas, Hasan Kandemir, Fatma Celik, Tuba Mutluer, Ahmet Buber, Mehmet Karadag, Nurdan Coban, Seyma Coskun, Zehra Hangul, Ebru Altintas, Ufuk Acikbas, Asli Giray, Yeliz Aka, Bilkay Basturk, Ozgur Kutuk

**Affiliations:** 1 Department of Child and Adolescent Psychiatry, Baskent University School of Medicine, Dr. Turgut Noyan Medical and Research Center, Adana, Turkey; 2 Department of Child and Adolescent Psychiatry, Bolu Abant Izzet, Baysal University School of Medicine, Bolu, Turkey; 3 Department of Child and Adolescent Psychiatry, Harran University School of Medicine, Sanliurfa, Turkey; 4 Department of Child and Adolescent Psychiatry, Private Practice, Istanbul, Turkey; 5 Department of Child and Adolescent Psychiatry, Mersin University School of Medicine, Mersin, Turkey; 6 Department of Child and Adolescent Psychiatry, Celal Bayar University School of Medicine, Manisa, Turkey; 7 Department of Psychology, Ankara Gazi Mustafa Kemal Occupational and Environmental Diseases Hospital, Ankara, Turkey; 8 Department of Child and Adolescent Psychiatry, Koc University School of Medicine, Istanbul, Turkey; 9 Department of Child and Adolescent Psychiatry, Pamukkale University School of Medicine, Denizli, Turkey; 10 Department of Child and Adolescent Psychiatry, Gaziantep University School of Medicine, Gaziantep, Turkey; 11 Department of Child and Adolescent Psychiatry, Adana City Training and Research Hospital, Adana, Turkey; 12 Department of Child and Adolescent Psychiatry, Private Practice, Adana, Turkey; 13 Department of Psychiatry, Baskent University School of Medicine, Dr. Turgut Noyan Medical and Research Center, Adana, Turkey; 14 Immunocore Ltd, Abingdon, Oxford UK; 15 Department of Genetics and Bioengineering, Alanya Alaaddin Keykubat University, Alanya, Turkey; 16 Department of Medical Biology, Baskent University School of Medicine, Ankara, Turkey; 17 Department of Immunology, Baskent University School of Medicine, Adana Dr. Turgut Noyan Medical and Research Center, Adana, Turkey; 18 Faculty of Engineering and Natural Sciences, Molecular Biology, Genetics and Bioengineering Program, Sabanci University, 34956 Tuzla, Istanbul, Turkey

**Keywords:** Tic disorders, Cytokines, Immune system, Inflammation, Tourette syndrome, Diseases, Psychiatric disorders, Quality of life, Risk factors

## Abstract

The etiology of tic disorders (TDs) is not precisely known, although several lines of evidence suggest involvement of the immune system in pathogenesis. Here, we aimed to determine the expression levels of pro-inflammatory and anti-inflammatory cytokines in children with TD and compare them with those of healthy controls. Furthermore, we also evaluated their association with clinical variables in the TD group. Within the study period, 88 children with tic disorders and 111 healthy control children were enrolled. Most children with tic disorders were diagnosed with Tourette’s disorder (n = 47, 53.4%) or persistent motor tic disorder (n = 39, 44.3%), while the remainder (n = 2, 2.3%) were diagnosed with persistent vocal tic disorder. We found that children with tic disorders had significantly elevated levels of IL-1β, TNF-α, IL-6 and IL-4 expression, while we detected lower expression levels of IL-17 in children with tic disorders. Our findings provide a molecular landscape of cytokine expression in children with TD, which may suggest a proinflammatory state not affected by the presence of comorbidity and symptom severity. Delineating the contribution of alterations in the immune system to the pathogenesis of tic disorders may pave the way for better therapeutic interventions.

## Introduction

Tic disorders in the DSM-5 include Tourette Syndrome (TS), persistent (chronic) motor/vocal tic disorder (CMVTD), and provisional tic disorder^1,2^. The consensus is that these disorders arise due to impaired function of the cortical-striatal-thalamic-cortical circuits, and aberrations of associated neurotransmitters may include dopamine, serotonin, gamma-aminobutyric acid (GABA), glutamate, acetylcholine, noradrenaline, and histamine^3–5^. The etiology of tic disorders is complex and involves multiple genetic, environmental, psychological, and immunological factors as well as their interactions^3–5^.

Tic disorders are characterized by high heritability and familial transmission. Candidate susceptibility genes included CNTNAP2, NLGN4, SLITRK1, HDC, IMMP2L, DRD2, DRD4, MAO-A, and GABAA receptors. These implicated potassium channels, membrane proteins, histidine metabolism, mitochondrial functioning, dopaminergic, GABAergic, and monoamine neurotransmitter function in etiology^3–6^. Genome wide association studies (GWAS) as well as studies evaluating single nucleotide polymorphisms (SNPs) suggest that tic disorders are polygenetic and the interaction between genetic predisposition and environmental factors increase the risk of their occurrence ^3–5,7^.

Studies on the environmental factors affecting the development of tic disorders have focused on prenatal, perinatal, and other developmental periods. Tic disorders in the offspring were found to be associated with maternal stress, nausea/vomiting, and smoking/alcohol/cannabis use during pregnancy. Low birth weight and a reduction in Apgar scores after birth may also increase risk. Familial and psychological factors were not found to be related to the onset of tics, but to their exacerbation. These factors may include parental psychopathology (especially maternal), poor parental relationships and family conflict ^3–6^.

Studies conducted over the last three decades have suggested that immune mechanisms may play a role in the development of TD. Various studies suggest that children with TD may have elevated levels of proinflammatory cytokines, such as TNF-α, IL-1β, IL-2, IL-6, IL-8, IL-12, IL-17, and IFN-γ, and the effect sizes for TNF-α and IL-6 seem to be especially large^8^. TD among children has been found to be associated with autoimmune disorders such as asthma, allergic rhinitis, allergic conjunctivitis, and possibly eczema and food allergies ^9^. Furthermore, TD and related syndromes have been shown to display abnormalities in microglial functioning ^10^. Consistent with the role of immune signaling in TD-related disorders, injection of soluble IL-2 and IL-6 receptors was found to induce motor stereotypies in rats, which correlated with their deposition in the striatum, thalamus, and various cortical regions ^11,12^. Exposure to maternal acute/chronic inflammation in utero has been found to be associated with various neurodevelopmental disorders, including TD, in offspring ^13^. Tic disorders are also known to arise after infections, as illustrated in cases of pediatric acute onset neuropsychiatric syndrome (PANS) ^14^. Moreover, dopamine itself has been shown to mediate the pathogenesis of TD and to exert immunomodulatory effects^15^.

Despite accumulating evidence of immune mechanisms in TD among children and adolescents, to the best of our knowledge, no study has evaluated cytokine expression levels among Turkish children with TD. A previous study from Turkey reported changes in cytokine levels in children with OCD^16^.

In this study, we examined the expression levels of IL-1β, IL-1α, IL-4, IL-6, IL-17, TNF-α, and TGF-β in peripheral blood mononuclear cells (PBMCs) of children with tics and healthy controls and determined the correlation between cytokine levels and clinical features to determine their contributions in children and adolescents with tic disorders.

## Materials and methods

### Study centers, sampling, and ethics

This study was conducted at the Child and Adolescent Psychiatry Departments from seven centers and involved patients followed up with TD diagnosis in these centers (persistent (chronic) motor or vocal tic disorder [307.22, ICD-10 F95.1] and Tourette’s disorder [307.23, ICD-10 F95.2] as per DSM-5)^1^. The diagnoses of the potentially eligible patients were corroborated through clinical interviews. The inclusion criterion for the TD group was a diagnosis of persistent motor or vocal tic disorder or Tourette’s disorder without known genetic syndromes and/or chronic neurological/medical conditions. Patients with intellectual disabilities (according to developmental history, mental status examination, and academic achievement), autism spectrum disorders, PANDAS/PANSS (Pediatric Autoimmune Neuropsychiatric Disorders Associated with Streptococci/Pediatric Acute Onset Neuropsychiatric Syndrome) and psychosis were excluded. Informed consent was obtained from the parents of the children included in the study, and the children provided verbal/written assent. The study was conducted in accordance with the Declaration of Helsinki and local laws and regulations. The study protocol was approved by the Institutional Review Board (IRB) of Inonu University (No:2014/ 236). The control participants were recruited from children coming for routine control visits to the departments of pediatrics in the study centers. They were matched in terms of gender and age to children with TD. The inclusion criterion for control children was a lack of lifetime psychopathology in the psychiatric interviews. The exclusion criteria for both children with TD and controls were acute respiratory or infectious disease; history of cardiovascular, endocrine, neurological, or other disorders/factors known to affect inflammatory indices; and history of receiving steroids and intravenous gamma globulin within the last 6 months prior to the study visit.

### Psychometric evaluations

The Children’s Depression Inventory (CDI) is a 27-item self-report scale developed to evaluate subjective symptoms of depression among 6 to 17-year-old children. The Turkish version was previously found to be valid and reliable. CDI was completed by children ≥ 8 years old in this study. Children scoring above 19 were accepted to display clinically significant depressive symptoms according to reliability and validity study^17,18^. The State-Trait Anxiety Inventory for Children (STAI-C) is a 40-item self-report scale developed to evaluate symptoms of state and trait anxiety ^19,20^. The STAI-C was completed by children ≥ 8 years in this study. Children scoring above the median on the STAI-C-Trait scale were considered to have significant trait anxiety. The Maudsley Obsessive Compulsive Symptom Checklist (MOCSL) is a 30-item self-report scale that evaluates subjectively reported obsessive symptoms ^21,22^. In this study, the MOCSL was completed by children aged ≥ 8 years. The Yale Global Tic Severity Scale (YGTSS) is a semi-structured clinician-applied interview that assesses the severity of tics in the past week. Motor and vocal tics were scored separately between 0 and 5 according to frequency, number, severity, complexity, and disability. Higher scores indicate greater severity. Finally, a clinical impairment score was added to form a total score ^23,24^.

### Sample collection, qRT‒PCR and ELISA assays

Ten milliliters of blood was collected into PAXGene Vacutainer tubes (Qiagen, Hilden, Germany), and RNA was isolated using the PAXGene Blood RNA Kit, as described by the manufacturer. RNA integrity and quality were confirmed using the Agilent 2100 Bioanalyzer (Agilent, Santa Clara, CA, USA). The mRNA expression profile of cytokines was determined using QuantiTect Primer assays as described before^25^. To evaluate the concordance between serum cytokine levels and mRNA expression data, serum samples from TD children and healthy control children were isolated and stored at − 80 °C. Serum concentrations of IL-4 (Catalog #: D4050), IL-1α (Catalog #: DLA50), IL-1β (Catalog #: DLB50), TGF-β (Catalog #: DB100B), IL-17 (Catalog #: D1700), IL-6 (Catalog #: D6050), and TNF-α (Catalog #: DTA00D) were measured using Quantikine enzyme-linked immunosorbent assays, according to the manufacturer’s protocol (R&D Systems, Minneapolis, MN, USA). Statistical analysis to evaluate the significance of serum cytokine levels in different groups was conducted using the Student’s *t*-test module and correlation analysis was performed using the non-parametric Spearman *r* correlation test with a two-sided *t*-test for significance in GraphPad Prism 10.0.2 software (San Diego, CA, USA). Values of *p* < 0.05, *p* < 0.01 and p < 0.001 were considered statistically significant.

### Statistical analyses

Data were entered into a database prepared with Statistical Program for Social Sciences (SPSS™, IBM Inc., Armonk, NY) Version 22.0. Analyses were conducted using SPSS and Jamovi (Jamovi Project, https://www.jamovi.org) version 2.3.21). Nominal data were summarized as counts and frequencies, while quantitative data were summarized as the means and standard deviations or medians and interquartile ranges (IQRs) depending on normality and outliers. Comparisons between groups were conducted with chi-square tests for nominal variables. Yates’, Fisher’s, and likelihood ratio corrections were used as needed. Bivariate correlations between symptom severities and cytokine expression levels were evaluated using Spearman correlation analyses. Multivariate analysis of variance (MANOVA) with follow-up ANOVA was used to evaluate the effects of diagnosis on cytokine expression levels. P was set at 0.05 (two-tailed). Bonferroni corrections were used to adjust for multiple comparisons (Gaetano J. Holm‒Bonferroni sequential correction: An Excel ™ calculator. 2018. https://www.researchgate.net/profile/Justin-Gaetano). Effect sizes for significant findings were also reported.

### Compliance with ethics guidelines

Meryem Ozlem Kutuk, Ali Evren Tufan, Fethiye Kilicaslan, Cem Gokcen, Gulen Guler Aksu, Cigdem Yektas, Hasan Kandemir, Fatma Celik, Tuba Mutluer, Ahmet Buber, Mehmet Karadag, Nurdan Coban, Seyma Coskun, Zehra Hangul, Ebru Altintas, Ufuk Acikbas, Aslı Giray, Yeliz Aka, Bilkay Basturk and Ozgur Kutuk declare that they have no conflict of interest. All authors have given approval for this version to be published. The study was approved by the appropriate institutional and/or national research ethics committee and the study was performed in accordance with the ethical standards as laid down in the 1964 Declaration of Helsinki and its later amendments or comparable ethical standards. Informed consent was obtained from all patients for being included in the study.

## Results

During the study period, 88 children with tic disorders (n = 64, 72.7% male) and 111 control children (n = 74, 66.7% male) were enrolled. The mean ages of the children with tic disorders and control children were 11.2 (SD = 3.1) and 12.0 (SD = 3.2) years, respectively. The groups did not differ significantly in terms of sex (χ2 = 0.6, p = 0.444, Yates’ correction) or mean age (t [197] = − 1.9, p = 0.063, Student’s t-test for independent groups). Other sociodemographic and familial variables of the children with tic disorders and control children are shown in Table 1.

**Table 1:** Sociodemographic and familial features of children with tic disorders and control children.

N (%)		Tic Disorder (n = 88)	Control (n = 111)	χ 2/dF or t	P*	E.S.**
Educational status	Not attending school	1 (1.1)	3 (2.7)	12.7/4	*0.013*	0.25
	Kindergarten/preschool	6 (6.8)	1 (0.9)			
	Primary school	34 (38.6)	26 (23.4)			
	Secondary school	28 (31.8)	51 (45.9)			
	High school	19 (21.6)	30 (27.0)			
Maternal age (years)		37.9 (6.7)	38.7 (7.1)	− 0.8/197	0.419	–
Maternal education	Primary school or lower	47 (53.4)	47 (42.7)	4.0/3	0.266	–
	Secondary school	10 (11.4)	9 (8.2)			
	High school	14 (15.9)	23 (20.9)			
	University or higher	17 (19.3)	31 (28.2)			
Maternal vocation	Housewife	70 (79.5)	75 (67.6)	7.8/4	0.167	–
	Worker	2 (2.3)	5 (4.5)			
	Civil servant	12 (13.6)	25 (22.5)			
	Artisan	3 (3.4)	4 (3.6)			
	Retired	1 (1.1)	0 (0.0)			
Paternal age (years)		42.0 (6.5)	41.3 (10.2)	0.6/197	0.581	–
Paternal education	Primary school or lower	29 (33.0)	32 (28.8)	0.8/3	0.841	–
	Secondary school	9 (10.2)	9 (8.1)			
	High school	23 (26.1)	32 (28.8)			
	University or higher	27 (30.7)	38 (34.2)			
Paternal vocation	Jobless	3 (3.4)	1 (0.9)	7.0/4	0.222	–
	Worker	23 (26.1)	23 (20.7)			
	Civil servant	27 (30.7)	36 (32.4)			
	Artisan	30 (34.1)	40 (36.0)			
	Retired	5 (5.7)	7 (6.3)			
Family status	Intact/Nuclear	85 (96.6)	97 (87.4)	5.3/1	*0.023*	*0.16*
	Separated/widowed/divorced	3 (3.4)	14 (12.6)			
Maternal psychopathology present		14 (15.9)	11 (9.9)	1.1/1	0.292	–
Paternal psychopathology present		7 (8.0)	6 (5.4)	0.2/1	0.664	–

*Chi square test with likelihood ratio, Yates’ corrections, Fisher’s exact test and t test for independent groups.

**Phi and Cramer’s V.

Healthy control children were significantly more likely to attend secondary and high schools than were children with tic disorders, while they were less likely to live in intact nuclear families. Apart from those, no significant differences emerged between groups. Most children with tic disorders were diagnosed with Tourette’s disorder (n = 47, 53.4%) or persistent motor tic disorder (n = 39, 44.3%), whereas the remainder (n = 2, 2.3%) were diagnosed with persistent vocal tic disorder. According to evaluations with the Yale Global Tic Disorder Severity Scale, mean scores for motor and vocal tics were 13.7 (SD = 4.9) and 5.2 (SD = 6.2), respectively. The mean global impairment and total scores were 29.2 (SD = 11.4) and 48.1 (SD = 18.6), respectively. Most children were classified as having either moderate (n = 40, 45.5%) or significant (n = 32, 36.4%) tics, whereas mild (n = 9, 10.2%) or severe (n = 7, 8.0%) tics were rare.

Additionally, most children had either one (n = 47, 53.4%) or two (n = 9, 10.2%) comorbid diagnoses (median = 1.0, IQR = 1.0). The most common comorbid diagnoses were attention deficit/hyperactivity disorder (ADHD, n = 38, 43.2%), anxiety disorder (n = 13, 14.8%), and obsessive–compulsive disorder (OCD, n = 8, 9.1%). Notably, one child each (1.1%) was diagnosed with trichotillomania, childhood-onset speech fluency disorder, enuresis, and oppositional defiant disorder. Most children were receiving pharmacological treatment (n = 72, 81.8%) with either one (n = 41, 46.6%) or two (n = 31, 35.2%) agents. The most common psychopharmacological treatments were aripiprazole (n = 42, 48.9%), methylphenidate (n = 15, 17.0%) and atomoxetine/risperidone/haloperidol (n = 12, 13.6%, each). Eight children (9.1%) were treated with SSRIs, while only one (1.1%) received olanzapine treatment. Self-reported symptoms of anxiety, depression, and obsessive–compulsive disorder among children with tic disorders and control children are listed in Table 2.

**Table 2:** Self-reported anxiety, depression, and obsessive–compulsive disorder symptoms among children with tic disorder and control children.

Median (IQR)	Tic Disorder (n = 88)	Control (n = 111)	Z	P*	E.S
State-trait anxiety inventory for children- state	33.0 (10.3)	31.0 (10.0)	− 1.8	0.072	0.13
State-trait anxiety inventory for children- trait	36.0 (12.0)	35.0 (10.0)	− 1.3	0.189	−
Children’s depression inventory	10.0 (10.0)	8.0 (8.0)	− 1.6	0.108	−
Maudsley obsessive compulsive symptom checklist	16.0 (9.0)	14.5 (10.0)	− 2.1	*0.036*	*14.9*

*E.S*. Effect Size.

*Mann‒Whitney U test.

Bivariate comparisons showed that children with tic disorders tended to have elevated levels of state anxiety and obsessive–compulsive symptom scores. Children with Tourette’s disorder, persistent motor, and vocal tic disorders did not differ significantly in terms of self-reported symptoms (Mann–Whitney U test, p > 0.05). Bivariate correlations of psychometric measures between the YGTSS- Total Tic Score and Global Impairment score revealed that only global impairment correlated significantly with the MOCSL score (Spearman’s rank order correlation rho = 0.003, rho’ = 0.021, Holm‒Bonferroni corrected). Less than half of the TD children had trait (n = 36, 40.9%) or state (n = 35, 39.8%) anxiety above the median, while only ten (11.4%) reported clinically significant depressive symptoms.

Finally, we compared children with tic disorders and control children in terms of cytokine expression (i.e., IL-1α, IL-1β, TNF-α, IL-6, TGF-β, IL-17, and IL-4) using qRT-PCR. Apart from TNF-α (p = 0.159) and IL-6 (p = 0.198) among children with tics and IL-17 among controls (p = 0.129, Kolmogorov‒Smirnov test with Lilliefors correction), none of the cytokines conformed to assumptions of normality. Covariance matrices of dependent variables were not equal across groups (Box’s M = 218.0, F = 7.5, p = 0.000), and only the error variances for IL-17 were equal (p = 0.393, Levene’s test). Therefore, interleukin levels across groups were compared using multivariate analysis of variance (MANOVA) with the Pillai’s trace method. According to MANOVA, the groups differed significantly in terms of interleukin levels (F [7.0, 191.0] = 13.2, p = 0.000, partial η2 = 0.33). The results of the univariate ANOVAs and interleukin levels across the groups are shown in Table 3. In addition, we evaluated the serum levels of cytokines in children with tic disorders and healthy controls using ELISA to evaluate the concordance of qPCR results with serum protein levels. As shown in Fig. S1, serum IL-1β, TNF-α, IL-6, and IL-4 levels were higher in children with tic disorders than in healthy controls, which is in line with the qRT-PCR data. Furthermore, we did not detect any significant changes in IL-1α and TGF-β levels, in concordance with the qRT-PCR results. These results may indicate that the increased expression of cytokines in children with tic disorders is a consequence of reprogrammed gene expression in PBMCs. Next, we sought to evaluate the correlation between upregulated cytokines (IL-1β, TNF-α, IL-6, and IL-4) in children with tic disorders. As demonstrated in Fig. S2, we found a significant correlation between serum IL-1β and TNF-α levels in children with tic disorders (****, p < 0.0001). However, we could not detect any correlation for IL-1β/IL-6, TNF-α/IL-6, IL-6/IL-4, IL-1β/ IL-4 or TNF-α/IL-4. Consistent with the ELISA data, we detected the same correlation pattern following analysis of qRT-PCR results (Fig. S2). Notably, examination of cases with concomitant upregulation of IL-1β and TNF-α levels did not reveal any distinctive clinical presentation. Although we could not detect a significant alteration in IL-17 serum levels, we observed a trend toward lower IL-17 serum levels in children with tic disorders (Fig. S2).

**Table 3:** mRNA expression levels of cytokines in children with tic disorders and healthy control children.

Mean (SD)	Tic Disorder (n = 88)	Control (n = 111)	F	P*	Partial η2
IL-1α	1.7 (0.8)	1.8 (0.7)	0.3	0.562	0.00
IL-1β	2.1 (1.2)	1.9 (0.8)	4.2	*0.042*	*0.02*
TNF-α	2.3 (1.1)	1.7 (0.7)	20.0	*0.000*	*0.09*
IL-6	3.1 (1.9)	1.7 (0.6)	55.2	*0.000*	*0.22*
TGF-β	1.7 (1.0)	1.7 (0.6)	0.00	0.961	0.00
IL-17	1.8 (0.9)	2.2 (0.8)	10.4	*0.001*	*0.05*
IL-4	2.0 (1.1)	1.6 (0.7)	10.1	*0.002*	*0.05*

mRNA expression levels were expressed as relative fold expression normalized to GAPDH.

*IL* interleukin, T*NF* tumor necrosis factor, *TGF* tumor growth factor.

*ANOVA with Bonferroni correction.

Hence, this finding suggests that cellular mechanisms other than transcriptional regulation may mediate the secretion and stability of IL-17.

According to univariate ANOVAs, children with tic disorders had significantly elevated levels of IL-1β, TNF-α, IL-6 and IL-4 expression, while controls had significantly elevated levels of IL-17 expression. The type of TD did not affect cytokine expression levels (Mann‒Whitney U test, p > 0.05). Among children with tic disorders, state anxiety scores were significantly and positively correlated with IL-17 levels (rho = 0.32, p = 0.006), whereas among control children, state anxiety correlated significantly and negatively with IL-6 levels (rho =− 0.25, p = 0.026). Moreover, self-reported obsessive–compulsive symptom scores were significantly and negatively correlated with IL-4 levels (rho = − 0.22, p = 0.049) among controls. In addition, no significant correlations emerged between interleukin levels and self-reported anxiety, depression, or obsessive–compulsive disorder symptoms. The YGTSS-total tic and impairment scores did not correlate significantly with cytokine levels. Lastly, we conducted a regression analysis with the total YGTS tic score as the dependent variable and IL-1β, TNFα, IL-6, IL-17, IL-4, number of comorbidities, and number of treatments as independent variables (Enter method). We entered the predictors in successive steps to evaluate their contributions to tic severity. The autocorrelation was within acceptable range (Durbin-Watson, DW = 1.5) and there were no problems with multicollinearity (variance inflation factor, VIF between 1.1–2.0). The final model (F = 34.7, p = 0.000) and the preceding model steps were all significant (F = 4.5–33.4, p < 0.05). Sole significant predictors of total tic score were IL-6, number of comorbidities, and number of treatments (Table 4).

**Table 4:** Regression analysis of Yale Global Tic Severity Total Tic Score with predictors.

	β	t	p	95% CI	VIF	p for F change
IL-1β	0.05	0.989	0.324	− 0.5–1.6	1.1	0.035
TNFα	0.01	0.191	0.849	− 1.1–1.3	1.1	0.001
IL-6	0.23	4.4	*0.000*	1.0–2.5	1.2	0.000
IL-17	0.02	0.4	0.677	− 1.0–1.5	1.1	0.067
IL-4	–0.02	− 0.4	0.669	− 1.5–1.0	1.1	0.454
Number of comorbidities	0.38	5.7	*0.000*	4.9–10.2	1.9	0.000
Number of treatments	0.32	4.6	*0.000*	2.7–6.6	2.0	0.000

R^2^ IL-1 β = 0.022, adjusted R^2^ TNFα = 0.071, adjusted R^2^ IL-6 = 0.223, adjusted R^2^ IL-17 = 0.232, adjusted R2 IL-4 = 0.230, adjusted R^2^ number of comorbidities = 0.496, adjusted R^2^ number of treatments = 0.544.

*CI* Confidence Interval, *VIF* Variance Inflation Factor.

## Discussion

This multicenter, cross-sectional, case–control study evaluated the expression levels of cytokines in the peripheral blood mononuclear cells of children diagnosed with Tic disorders and compared them with controls. Complex neurobiological and genetic mechanisms, alteration of iron metabolism, and environmental factors are thought to interact with each other in the etiology of TD, and immune dysfunction may be a promising avenue of research in the onset and development of tic disorders^8–10,14^. In our study, children with tic disorders had significantly elevated levels of IL-1β, TNF-α, IL-6 and IL-4 expression, while controls had significantly elevated levels of IL-17 expression. There were no significant differences in cytokine levels between the TD groups. Among children with tic disorders, state anxiety scores were significantly and positively correlated with IL-17 expression levels. In the regression analysis, only IL-6 significantly predicted higher tic severity.

The gender ratios of children with TD in our sample, their mean age and features of comorbid diagnoses conform to those reported in previous studies^1,2,6^. Almost half of our sample group was prescribed aripiprazole, while more than four-fifths were receiving psychopharmacological treatment for either TD or comorbid conditions^26^. Cytokines are multifunctional pleiotropic proteins that play a crucial role in cell-to-cell communication and cellular activation. In addition, many cytokines can act as neuromodulators, and their expression is regulated in the peripheral and central nervous systems by means of neurotransmitters^27^. A recent meta-analysis found increased levels of proinflammatory cytokines in pediatric patients with Tourette syndrome, including TNF-α and IL-6^8^. Consistent with this analysis, we found elevated levels of IL-1β, TNF-α, IL-6, and IL-4 expression among children with TD, whereas state anxiety among those children correlated positively with IL-17 levels. In regression analysis, IL-6 was the sole significant predictor of tic severity. Among these cytokines, IL-1β, TNF-α, IL-6, and IL-17 are pro-inflammatory, whereas IL-4 is mainly anti-inflammatory. IL-6 is produced by most cell lines in the CNS and is neurotrophic for midbrain dopaminergic neurons, as well as cholinergic neurons in the basal forebrain and septum. It increases dopaminergic and serotonergic activity in the hippocampus and prefrontal cortex, regulates neuronal excitability and sleep, and elevated levels have been reported in various psychopathologies and neurodevelopmental disorders, including TD^28^. IL-6 levels are also known to be reduced after treatment with antipsychotics^29^. In fact, Tao et al. reported increased IL-6 serum concentrations in children with TD compared with the control group, which included 1724 patients and 550 healthy control subjects^30^. Studies have also reported lower or unchanged levels of IL-4, IL-10, TNF-α, and IL-6 among children with TD compared to controls^31–34^. The inconsistency in these studies may be due to different methodologies used to determine serum cytokine levels, medications, presence of comorbidities, maternal autoimmunity, or age of patients^31–35^.

TNF-α regulates the activation of microglia and astrocytes, regulation of blood–brain barrier permeability, glutamatergic neurotransmission, and synaptic plasticity^36^. Preclinical studies have suggested that it may affect serotonergic and dopaminergic neurotransmission^37^. An increase in TNF-α levels may increase the permeability of the blood‒brain barrier and lead to an enhanced autoimmune response, along with greater dopamine release in the basal ganglia, potentially contributing to the clinical symptoms of TS and related disorders^38–40^. Notably, TNF-α and IL-6 levels were significantly increased in the peripheral blood of patients with Tourette’s in a recent meta-analysis^5^.

IL-17 is a proinflammatory cytokine that acts synergistically with other proinflammatory cytokines and plays a role in neurotoxicity and autoimmunity^41^. Increased IL-17 levels have been reported in neurodegenerative, neurodevelopmental, and psychiatric disorders^42–44^. Elevated IL-17 levels have been reported to cause cell loss in dopaminergic neurons, and dopaminergic neurotransmission was found to modulate IL-17 secretion^45^. Surprisingly, we found reduced IL-17 expression in children with tic disorders compared with healthy control subjects. This may be due to the presence of acute infections (especially mucosal) in some of the control children or may reflect the effects of diet and vitamin D status^46,47^. Further studies on IL-17 levels among children with TDs may be conducted by controlling for these variables.

IL-4 is an anti-inflammatory cytokine that has paradoxical effects on the central nervous system. It may protect neurons against sepsis, ischemia, and multiple sclerosis while contributing to the degeneration of dopaminergic neurons in the substantia nigra in the presence of inflammation^48^. Moreover, IL-4 levels may play a role in synaptic homeostasis, and exposure to elevated levels in utero may increase the risk of neurodevelopmental disorders in the fetus^49^. TGF-β is an immunosuppressive cytokine that plays a role in neurodevelopment and synapse formation^50^. It has been reported that a lack of TGF-β leads to disorganization of the extracellular matrix, neurodegeneration, microgliosis, reductions in synaptophysin, and defects in glutamatergic and GABAergic synapses^51^. However, overexpression of TGF-β was also found to disrupt the extracellular matrix and lead to seizures/motor incoordination and behavioral abnormalities^52^. Although TGF-β overexpression has been demonstrated to induce different behavioral consequences depending on developmental stage, children with tic disorders in our study did not display changes in TGF-β expression levels compared to healthy controls. Finally, our results do not support the role of IL-4 in tic disorders.

Our results may support the presence of a pro-inflammatory status among Turkish children with TD. The lack of effects of comorbidities and symptom severity on cytokine levels may be due to the limited sample size. In addition, the limited number of children with mild or severe symptoms may have affected our ability to evaluate the relationship between symptom severity and cytokine levels. Our data should be evaluated within their limitations. First, our results may be valid only for patients with Tourette’s Disorder and Persistent Motor Tic Disorder receiving treatment at the study centers and those without comorbid chronic neurological/medical conditions. Children with persistent vocal tic disorder were also limited in our sample, and future studies should enroll more patients with this diagnosis. Second, we excluded patients with comorbid intellectual disabilities or autism spectrum disorders, which may have affected our results. Third, we evaluated intellectual disability in our sample according to psychiatric interviews, developmental history, academic achievement, and mental status examination, and evaluation of intellectual functioning with valid psychometric measures (e.g., Wechsler Intelligence Scale for Children-Fourth Edition, WISC-IV) may have enriched our results. Fourth, most of our sample was receiving treatment, especially antipsychotics, and had comorbid diagnoses that may have affected the cytokine levels. Fifth, evaluation of depression, anxiety, and obsessive–compulsive symptoms with clinician-rated measures, in addition to personal reports, may prove more valuable. Sixth, self-reports of depression, anxiety, and obsessive–compulsive symptoms may be subject to reporting and recall bias, in addition to shared method variance. Seventh, rather than unstructured clinical interviews, semi-structured interviews (e.g., K-SADS-PL Turkish version) may be preferable in clinical evaluations. Finally, in addition to cytokine levels, other measures of inflammation may be included to provide a more comprehensive battery.

Regardless of these limitations, our results support the presence of proinflammatory status among Turkish children with TD. Future studies should enroll children with diverse age ranges and symptom severities to evaluate the inflammatory status of Turkish children with TD. In conclusion, our study adds to the growing evidence supporting the importance of the immune system in the pathophysiology of TS and its related disorders. Recent research is progressively unveiling a complex interplay between neural transmission, immune response regulation and endocrine systems, which might provide further insight into the natural history of TS and its relationships with environmental stressors, especially infections^5,6,8–10^.

The cross-sectional and correlational nature of our study design precludes hypotheses of causality. However, considering the role of dopamine in the modulation of immune cells via autocrine/paracrine routes, as well as the expression of dopamine receptors on lymphocytes, prospective longitudinal studies may evaluate the role of the hypothesized hyperdopaminergic state of children with TD on their immune responses. The role of aberrant immunity in the brain development of children with neurodevelopmental disorders, including TD, awaits further study. This avenue of research requires the development of more valid animal models, as well as a larger availability of brain specimens. Once the complex interplay between neurodevelopment, immunity, and environmental stressors is elucidated in more detail, the effects of immunotherapy with antibodies or antibody components targeting specific cytokines (e.g., IL-6) may be evaluated in preclinical models. These potential therapies may prevent peripheral cytokine receptors from crossing the blood–‒brain barrier and prevent the induction of inflammatory cascades in the periphery with downstream effects. Additionally, the effects of anti-inflammatory agents such as cyclooxygenase inhibitors or n-acetylcysteine may be evaluated as potential treatments for TD.

### Supplementary Information

Supplementary Legends.

Supplementary Figure 1.

Supplementary Figure 2.

## Data Availability

The datasets used and/or analyzed during the current study available from the corresponding author on reasonable request.
