# Supplementary material for: Cytokine expression profiles in children and adolescents with tic disorders
Source: Sci Rep. 2024 Jul 2;14:15101. doi: 10.1038/s41598-024-62121-z (PMC11219894; doi:10.1038/s41598-024-62121-z)
Supplement: Supplementary file 1 — Supplementary Legends. [file 41598_2024_62121_MOESM1_ESM.docx]

**Supplementary Figure Legends**

**Supplementary Figure S1.** Altered serum levels of cytokines in Turkish children with tic disorders (TD) and healthy controls. Serum samples from children with TD and healthy control children were evaluated by ELISA. The results are representative of 3 independent measurements and expressed as pg/mL or ng/mL, accordingly. ***, p<0.001, unpaired, two-tailed Student’s t test.

**Supplementary Figure S2.** Correlation of TNF-α/ IL-1β, IL-1β/IL-6, TNF-α/IL-6, IL-6/IL-4, IL-1β/ IL-4 and TNF-α/IL-4 serum protein and mRNA expression levels was determined by non-parametric Spearman *r* correlation test (****P<0.0001, by two-tailed t test).
