# Supplementary figures and images for: Cytokine expression profiles in children and adolescents with tic disorders
Source: Sci Rep. 2024 Jul 2;14:15101. doi: 10.1038/s41598-024-62121-z (PMC11219894; doi:10.1038/s41598-024-62121-z)

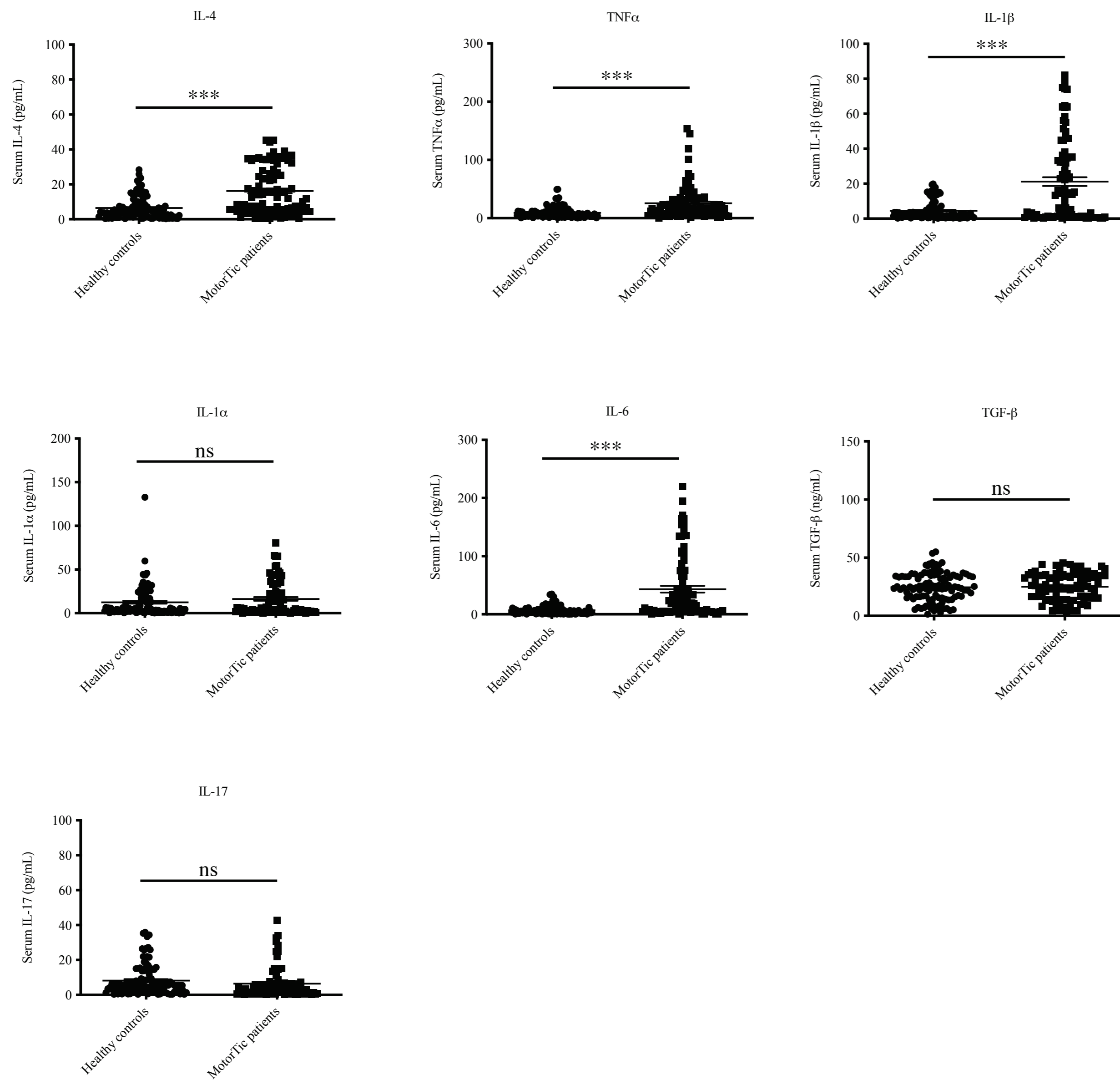

Supplement: Supplementary file 2 — Supplementary Figure 1. [file 41598_2024_62121_MOESM2_ESM.pdf]
