# Supplementary material for: Cytokine expression profiles in children and adolescents with tic disorders
Source: Sci Rep. 2024 Jul 2;14:15101. doi: 10.1038/s41598-024-62121-z (PMC11219894; doi:10.1038/s41598-024-62121-z)
Supplement: Supplementary file 3 — Supplementary Figure 2. [file 41598_2024_62121_MOESM3_ESM.pdf]

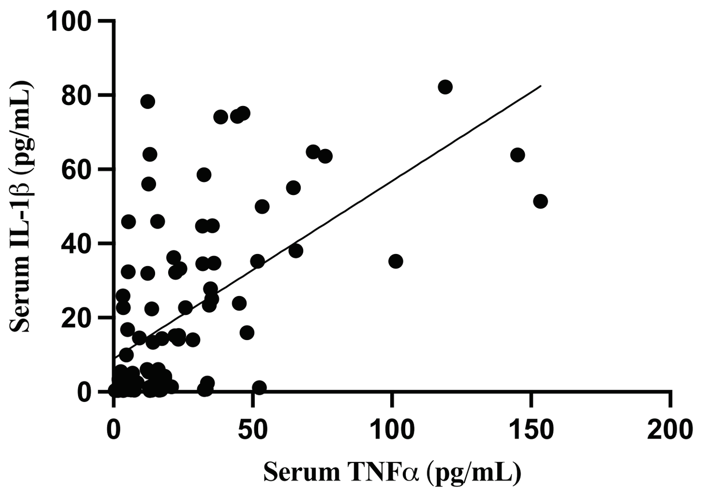

	TNF-alpha vs. IL-1beta
Spearman r	
r	0.5721
95% confidence interval	0.4068 to 0.7011
P value	
P (two-tailed)	<0.0001
P value summary	****
Exact or approximate P value?	Approximate
Significant? (alpha = 0.05)	Yes

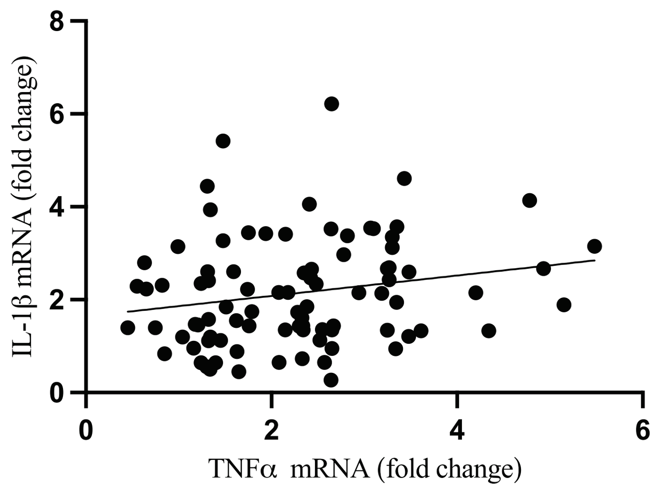

	TNF-alpha vs. IL-1beta
Spearman r	
r	0.2181
95% confidence interval	0.002769 to 0.4141
P value	
P (two-tailed)	0.0412
P value summary	*
Exact or approximate P value?	Approximate
Significant? (alpha = 0.05)	Yes

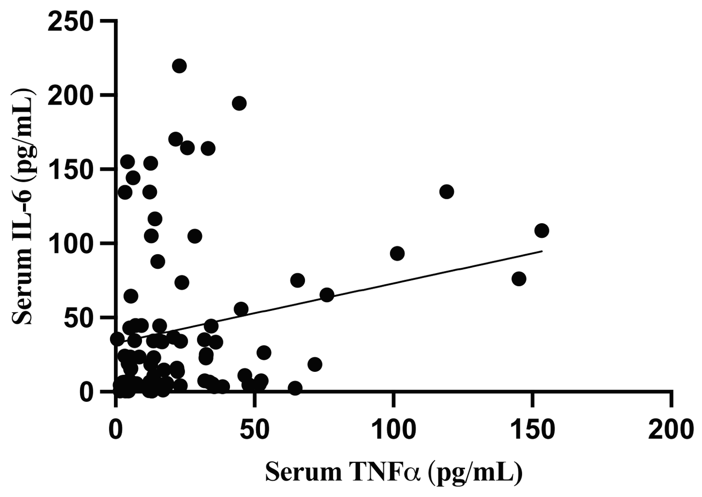

	TNF-alpha vs. IL-6
Spearman r	
r	0.1790
95% confidence interval	-0.03791 to 0.3798
P value	
P (two-tailed)	0.0952
P value summary	ns
Exact or approximate P value?	Approximate
Significant? (alpha = 0.05)	No

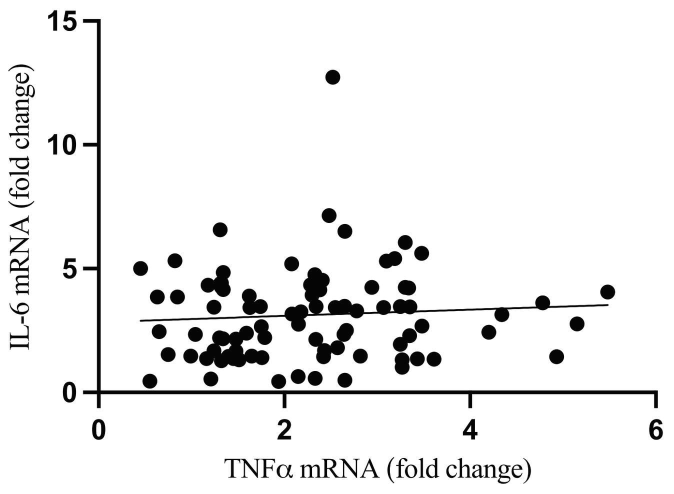

	TNF-alpha vs. IL-6
Spearman r	
r	0.06764
95% confidence interval	-0.1500 to 0.2790
P value	
P (two-tailed)	0.5312
P value summary	ns
Exact or approximate P value?	Approximate
Significant? (alpha = 0.05)	No

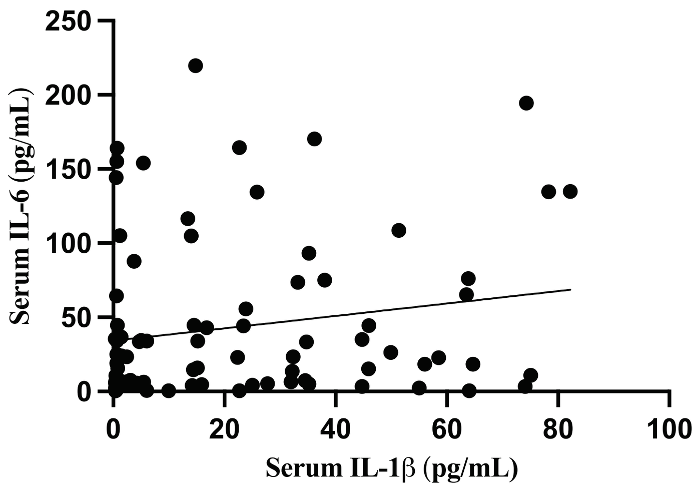

	IL-1beta vs. IL-6
Spearman r	
r	0.1947
95% confidence interval	-0.02168 to 0.3936
P value	
P (two-tailed)	0.0691
P value summary	ns
Exact or approximate P value?	Approximate
Significant? (alpha = 0.05)	No

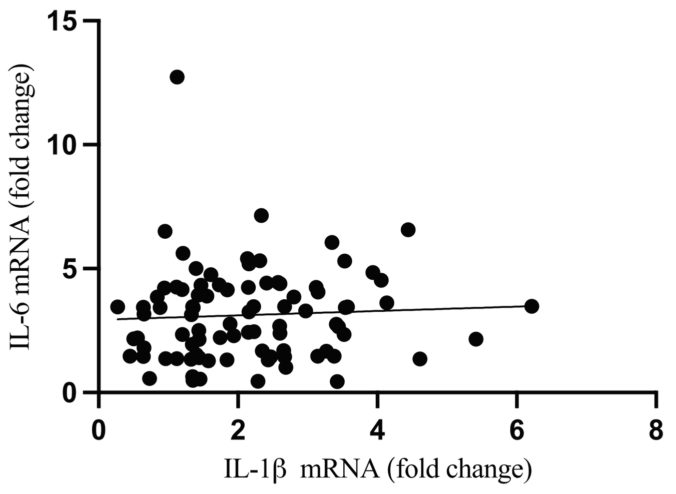

	IL-1beta vs. IL-6
Spearman r	
r	0.1056
95% confidence interval	-0.1124 to 0.3139
P value	
P (two-tailed)	0.3274
P value summary	ns
Exact or approximate P value?	Approximate
Significant? (alpha = 0.05)	No

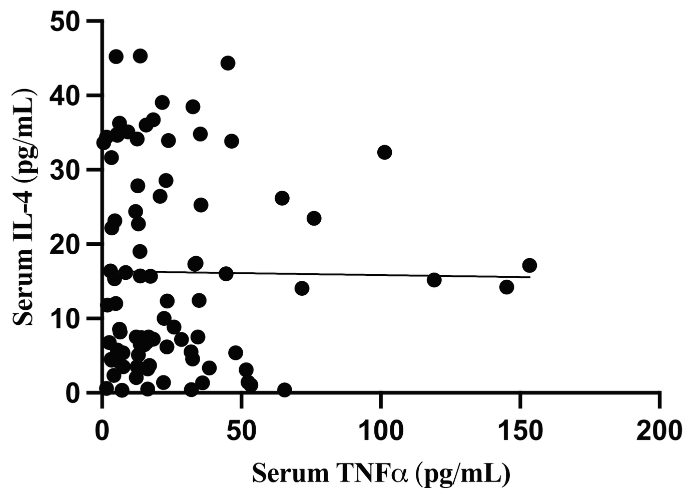

	TNF-alpha vs. IL-4
Spearman r	
r	-0.04573
95% confidence interval	-0.2586 to 0.1714
P value	
P (two-tailed)	0.6722
P value summary	ns
Exact or approximate P value?	Approximate
Significant? (alpha = 0.05)	No

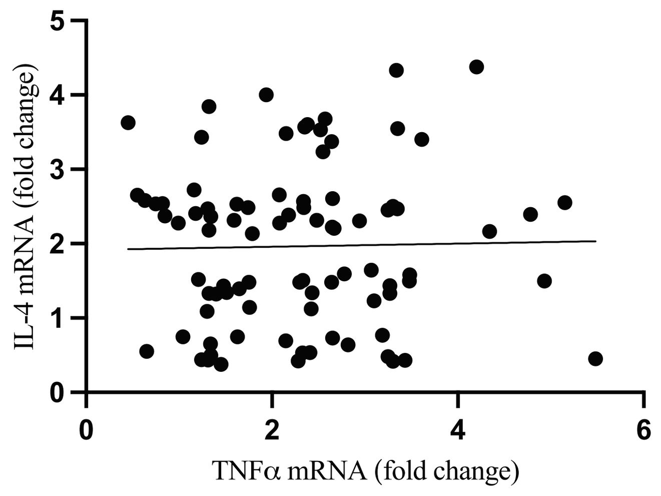

	TNF-alpha vs. IL-4
Spearman r	
r	0.002840
95% confidence interval	-0.2127 to 0.2181
P value	
P (two-tailed)	0.9790
P value summary	ns
Exact or approximate P value?	Approximate
Significant? (alpha = 0.05)	No

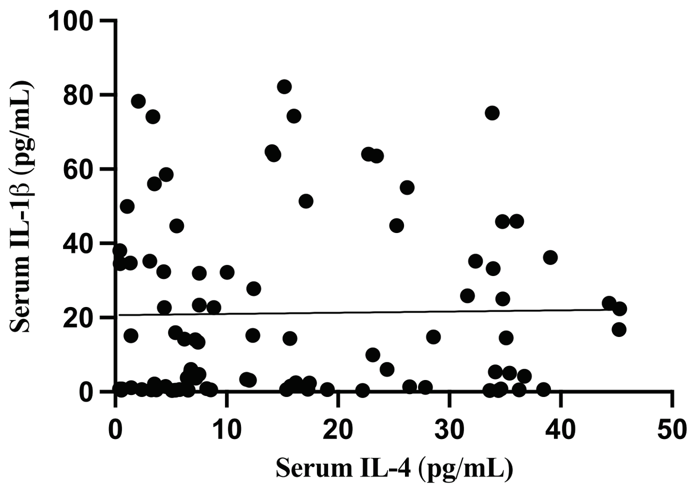

	IL-4 vs. IL-1beta
Spearman r	
r	0.04510
95% confidence interval	-0.1720 to 0.2580
P value	
P (two-tailed)	0.6765
P value summary	ns
Exact or approximate P value?	Approximate
Significant? (alpha = 0.05)	No

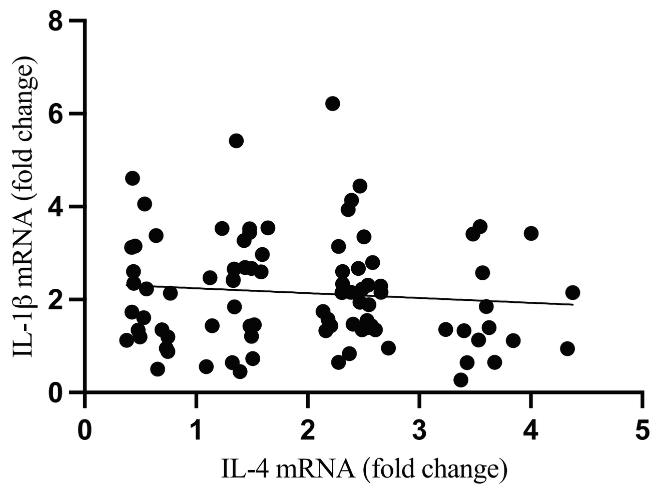

	IL-4 vs. IL-1beta
Spearman r	
r	-0.08800
95% confidence interval	-0.2978 to 0.1299
P value	
P (two-tailed)	0.4149
P value summary	ns
Exact or approximate P value?	Approximate
Significant? (alpha = 0.05)	No

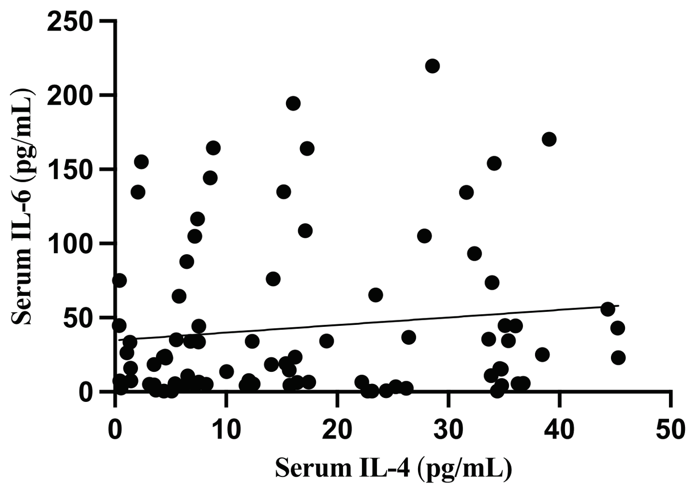

	IL-4 vs. IL-6
Spearman r	
r	0.1618
95% confidence interval	-0.05558 to 0.3645
P value	
P (two-tailed)	0.1320
P value summary	ns
Exact or approximate P value?	Approximate
Significant? (alpha = 0.05)	No

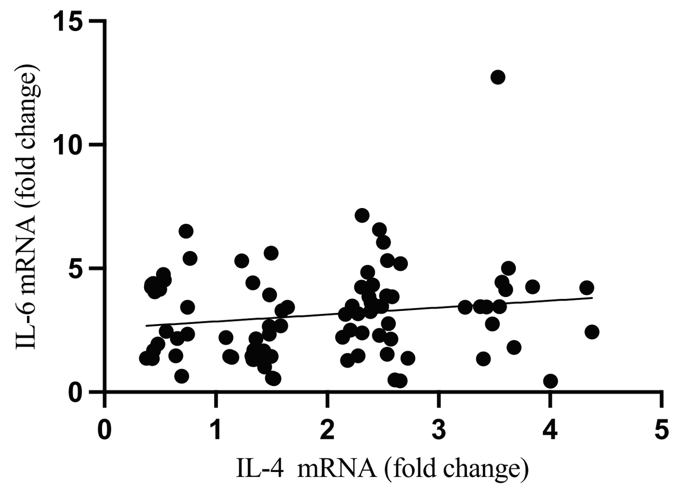

	IL-4 vs. IL-6
Spearman r	
r	0.1221
95% confidence interval	-0.09582 to 0.3289
P value	
P (two-tailed)	0.2569
P value summary	ns
Exact or approximate P value?	Approximate
Significant? (alpha = 0.05)	No

Supplementary Figure 2
